# Case report: Treatment with Phesgo® in a patient receiving hemodialysis

**DOI:** 10.3389/fonc.2024.1348343

**Published:** 2024-05-07

**Authors:** Catarina Pulido, Joana Albuquerque, Jorge Correia, José Luís Passos-Coelho

**Affiliations:** Oncology Department, Hospital da Luz Lisboa, Lisbon, Portugal

**Keywords:** case report, Phesgo, hemodialysis, advanced breast cancer, HER2-positive

## Abstract

**Introduction:**

Patients with metastatic HER2-positive breast cancer have multiple therapeutic options. However, most are not studied in the renal replacement therapy (RRT) setting.

**Case report:**

We report the use of Phesgo® (subcutaneous fixed-dose combination of trastuzumab and pertuzumab) combined with exemestane as a first-line treatment of metastatic HER2-positive breast cancer in a hemodialysis patient with multiple comorbidities. Partial response was attained, with disease progression after 8 months without evidence of significant toxicity.

**Discussions:**

This case report is, to our knowledge, the first published case documenting the use of Phesgo® in a hemodialysis patient. No new safety signs were seen, and activity was documented, adding support to the use of this drug combination in such a patient population.

## 1 Introduction

Breast cancer is the most frequent malignancy worldwide, responsible for a great burden of disease among women with multiple chronic conditions, including end-stage renal disease (ESRD). The improvement in survival of patients treated with renal replacement therapy (RRT) increases the chance of cancer development, with a higher incidence of breast cancer being reported in this population (1, 2).

Chronic kidney disease (CKD) does not limit surgical treatment or radiotherapy but affects the pharmacokinetics (PK) of drugs used as systemic treatment, increasing the risk of toxicity and making efficacy less predictable. These patients are usually excluded from clinical trials, and the available evidence on treatment efficacy and tolerance comes from case reports. Regarding the use of trastuzumab, no significant changes in PK were found in the RRT setting, with similar outcomes independent of drug formulation (intravenous or subcutaneous) (3–5). However, for pertuzumab, a different anti-HER2 monoclonal antibody, there are limited data on PK, and consequently, there is no recommendation for administering this drug to patients on RRT. To our knowledge, the only case report of a patient on hemodialysis treated with trastuzumab and pertuzumab used the intravenous formulation and documented the known and manageable toxicity profile without new efficacy concerns (6).

Herein, we report a further case of a patient on RRT treated with trastuzumab and pertuzumab, adding the first published report of the use of Phesgo® (a subcutaneous fixed-dose combination of pertuzumab and trastuzumab) in this population. Written informed consent was obtained from the participant/patient(s) for the publication of this case report.

## 2 Case report

### 2.1 Patient information

We report the case of a 52-year-old woman, autonomous and professionally active, without family or social support, with an Eastern Cooperative Oncology Group Performance Status (ECOG-PS) of 1. She noted a lump in her right breast and was diagnosed with breast cancer in 2019: invasive carcinoma of the breast, of no special type, G2, estrogen receptor (ER)-positive (90%), progesterone receptor-negative, HER2 3+, Ki67 60%, staged as cT3(m)N3M0—prognostic stage group IIIB.

Her medical family history included maternal breast cancer at 68 years old and paternal CKD. The patient had multiple comorbidities, namely, CKD. When she was 9 years old, she was diagnosed with Fanconi syndrome. She has been on hemodialysis three times a week (medium 3.7 hours per session) since 1998. From 1982 to 1990, the patient was on hemodialysis as well, having received a transplant in 1990 that failed in 1998. At the time of the first oncology consultation, the patient presented a left arm aneurysm of the arteriovenous fistula, secondary hyperparathyroidism, secondary hypertension, severe pulmonary hypertension, uremic neuropathy, malnutrition, chronic hepatitis C virus infection genotype 2, prior acute colonic diverticulitis complicated with abscess with surgical management and colostomy, major depression, and status post-thyroidectomy and right neck dissection (May 2017) for papillary thyroid carcinoma.

### 2.2 Clinical findings

The patient was asthenic, and her physical exam denoted malnutrition, a right breast lesion measuring 10 × 7 cm, right axilla and right supra- and infraclavicular lymphadenopathies, and right arm lymphedema. The remaining physical exam found no evidence of breast cancer metastatic disease (cM0).

### 2.3 Timeline

**Figure 1:**
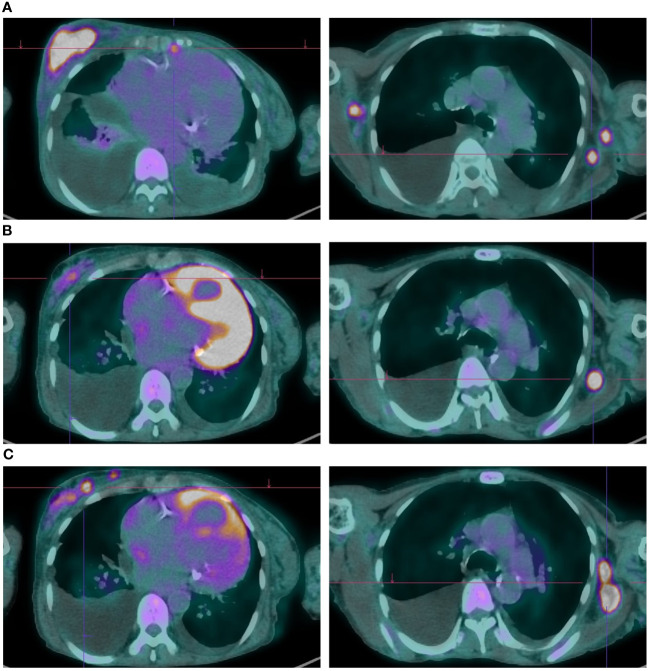
Metabolic and radiologic evaluation on FDG-PET of cancer lesions throughout Phesgo® treatment. **(A)** Obtained on July 22, 2022; showed disease progression with an increased atypical lesion in the right breast and bilateral lymphadenopathies. **(B)** Captured on January 10, 2023; demonstrated a favorable response to Phesgo**®** treatment with reduced breast lesion size and improved lymphadenopathies. **(C)** Obtained on April 20, 2023; indicated disease progression with new hypermetabolic foci, pleural effusion, ascites, and lytic lesions.

Given the diagnosis of locally advanced HER2-positive, ER-positive breast cancer, along with the patient’s comorbidities and preferences (prioritizing tolerability and flatly refusing chemotherapy), neoadjuvant therapy with subcutaneous trastuzumab and oral tamoxifen was started in January 2020. The best response was a partial response with good tolerance and no cardiac toxicity. After 6 months of therapy, and repeatedly thereafter, curative surgery was proposed, which the patient refused, maintaining trastuzumab and hormonal therapy up until July 2022, when after 30 months of therapy, locoregional, skin, pleural, peritoneal, and lymph node progression was confirmed (**Figure 1A**). During this period (until July 2022), the case was rediscussed in the tumor board, and alternatives such as radiotherapy were discussed and dismissed considering it would not be curative by itself nor effective palliating the lymphedema—the only local symptom the patient presented at that point.

### 2.4 Diagnostic assessment

At the tumor board and considering the advice of the patient’s nephrologist, first-line treatment with paclitaxel, trastuzumab, and pertuzumab was recommended.

### 2.5 Therapeutic interventions

The patient refused chemotherapy, so she was started on Phesgo® combined with exemestane in August 2022 (by then, the patient was postmenopausal). Phesgo® was administered every 21 days, 24 hours after the last hemodialysis session and 24 hours before the next.

### 2.6 Follow-up and outcomes

**Figure 2:**
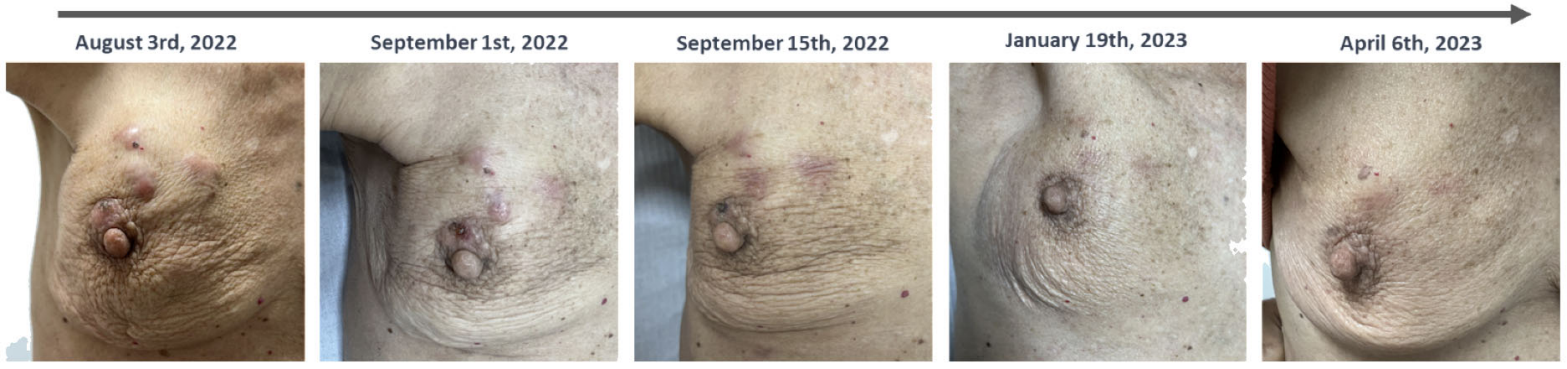
Temporal evolution of clinical breast and skin lesions. A favorable clinical response was evident following the initiation of Phesgo in August 2022, with complete resolution of skin lesions observed by January 2023. However, upon completing 10 cycles of Phesgo by April 2023, there was a recurrence of skin lesions, concurrent with an increase in the size of the breast lesion and the identification of new left axillary lymphadenopathies.

Three weeks after starting Phesgo® and exemestane, a reduction in the dimensions of the breast and skin lesions was already noted (**Figure 2**).

In the first response evaluation by positron emission tomography (PET) with fluorine-18 fluorodeoxyglucose (FDG), a partial metabolic response was documented with a clear decrease in the uptake in the breast lesion, the lymph nodes, and the pleural nodules, in addition to a decrease in the pleural effusion volume (**Figure 1B**). In January 2023, all the skin lesions had disappeared.

No adverse events were noted, and the left ventricle ejection fraction and strain remained within the normal range (**Table 1**).

**Table 1:** Temporal evolution of left ventricular ejection fraction and strain.

Date	GLS	LVEF
May 2022	−15.8%	53%
September 2022	−15.7%	50%
January 2023	–	50%
April 2023	−16.3%	53%

The table illustrates the temporal evolution of left ventricular ejection fraction (LVEF) and global longitudinal strain (GLS) documenting normal values for both parameters throughout the recorded period.

The patient completed 10 cycles of Phesgo® until April 2023, when some of the skin lesions reappeared, the breast lesion increased in size, and new left axillary lymph nodes were identified (**Figure 2**). FDG-PET in April 2023 documented disease progression in the breast, pleura, peritoneum, and lymph nodes (**Figure 1C**). The patient maintained an ECOG-PS of 1 and started a second-line anti-HER2 therapy recommended by the tumor board.

### 2.7 Discussion

Our report is, to our best knowledge, the first published case report of Phesgo® treatment in a patient on hemodialysis. The choice of Phesgo®, a subcutaneous fixed-dose formulation of pertuzumab and trastuzumab, instead of the intravenous formulation was justified by the right arm lymphedema, the left arm aneurysm of the arteriovenous fistula, and the limited vascular access options. Also, according to PHranceSCa trial results, most patients prefer the subcutaneous formulation, with the most common reasons being “less time in the clinic” and “more comfortable during administration”, which were very important to this professionally active patient, already spending much time in the hemodialysis center (7). In the absence of dose and schedule recommendations, we chose to administer Phesgo® on a different day from dialysis, which was conducted at another institution. Due to pertuzumab’s high molecular weight (148 kDa), removal of Phesgo® by hemodialysis was not expected (8). Despite prior reports suggesting that a lower glomerular filtration rate could be associated with an increased risk of cardiotoxicity for both trastuzumab and pertuzumab, our patient did not experience cardiac toxicity. Considering the limited safety and dosage data for aromatase inhibitors (AI) in patients with a glomerular filtration rate under 30 mL/min, we chose exemestane due to its minimal renal elimination (<1%) compared to other options (anastrozole 11%, letrozole 90%, and fulvestrant 8%) (9). In the case presented, Phesgo progression-free survival (PFS) was 8 months, lower than reported in the phase II PERTAIN trial, which compared the combination of trastuzumab plus pertuzumab plus an AI with trastuzumab plus an AI, with a median PFS of 20.6 *vs.* 15.8 months [hazard ratio (HR) 0.67, p = 0.006]. While these results are encouraging for a chemotherapy-free regimen, it must be noted that one-half of patients received induction chemotherapy with a taxane prior to switching to maintenance exemestane. Furthermore, patients with CKD or other comorbidities and patients with disease progression on prior trastuzumab were excluded from the trial, which may explain the lower PFS observed in this case (10–12). One limitation of this report is the absence of pharmacokinetic data, which were not collected considering that they would not lead to therapy modification (in the absence of guidance for that) but would add morbidity and costs to the patient’s care.

To conclude, our report demonstrates the safe and effective use of the association of trastuzumab plus pertuzumab in a patient with end-stage renal disease undergoing RRT and raises no red flags to the administration of the subcutaneous fixed-dose formulation in this setting. More studies are needed to assess the PK of anticancer drugs in patients on RRT and its efficacy and security in a real-world population. Meanwhile, some important lessons can be taken from this case, such as the importance of a multidisciplinary approach including medical subspecialties, in this case, nephrology. This was key to allowing the patient access to innovative oncological treatment despite her comorbidities and in accordance with her preferences. Raising awareness and special recommendations are needed for this patient’s subgroup not only to guide drug use and dosing but also to preclude undertreatment, especially in the curative setting.

## 3 Patient perspective

The treatment was well tolerated and administered in a comfortable schedule, permitting the maintenance of hemodialysis and most of the planned daily activities. The response in the breast was significant, with regression of the main lesions. This treatment is a valid option for disease control in patients with advanced disease (unresectable locally advanced or metastatic) and multiple comorbidities who cannot tolerate and/or refuse chemotherapy after being duly informed. In oncology and especially in the palliative setting, patient preferences are important and should be considered in shared decision-making. Information on disease course and treatment recommendations must be tailored to the individual patient and account for his/her preferences since some treatments may impact an outcome such as survival at the cost of a decrease in quality of life or patient independence.

## Data availability statement

The original contributions presented in the study are included in the article. Further inquiries can be directed to the corresponding author.

## Ethics statement

Written informed consent was obtained from the patient for the publication of any potentially identifiable images or data included in this article.

## Author contributions

CP: Conceptualization, Funding acquisition, Methodology, Resources, Supervision, Validation, Writing – original draft, Writing – review & editing. JA: Conceptualization, Methodology, Resources, Validation, Writing – original draft, Writing – review & editing. JC: Conceptualization, Methodology, Resources, Validation, Writing – original draft, Writing – review & editing. JP-C: Conceptualization, Methodology, Resources, Validation, Writing – original draft, Writing – review & editing.
